# Knockout of interleukin-17A protects against sepsis-associated acute kidney injury

**DOI:** 10.1186/s13613-016-0157-1

**Published:** 2016-06-22

**Authors:** Cong-juan Luo, Feng Luo, Li Zhang, Yan Xu, Guang-yan Cai, Bo Fu, Zhe Feng, Xue-feng Sun, Xiang-mei Chen

**Affiliations:** Department of Nephrology, The Affiliated Hospital of Qingdao University, Shandong, 266003 People’s Republic of China; State Key Laboratory of Kidney Diseases, Department of Nephrology, Chinese PLA General Hospital and Medical School of Chinese PLA, No.28 Fuxing Road, Beijing, 100853 People’s Republic of China; Department of Cardiology, Liaocheng People’s Hospital, Shandong, 252000 People’s Republic of China

**Keywords:** IL-17A knockout, Sepsis, Sepsis-associated acute kidney injury

## Abstract

**Background:**

Sepsis-associated acute kidney injury (SA-AKI) is an independent risk factor for death in patients with sepsis, but treatment for it is limited. To improve the diagnosis and treatment of SA-AKI, we must first understand its pathogenesis. Recently, interleukin (IL)-17A has been shown to be associated with the pathogenesis of acute kidney injury and sepsis, but its role in SA-AKI remains unclear.

**Methods:**

SA-AKI was induced in male C57BL/6 and IL-17A^−/−^ mice using cecal ligation and puncture (CLP) operations for 24 h.

**Results:**

At 7 days, only seven mice survived in the wild-type septic group, but nine survived in the IL-17A^−/−^ septic group, corresponding to survival rates of 25 % and 45 %, respectively. At 24 h after CLP operations, both wild-type and IL-17A^−/−^ septic mice developed kidney injury. The IL-17A^−/−^ septic mice exhibited decreased serum creatinine and blood urea nitrogen levels and an improved acute tubular necrosis score. The IL-17A^−/−^ septic mice exhibited decreased IL-6, interferon-γ, tumor necrosis factor-α, CXCL1, CXCL2, and CXCL5 expression in kidney tissue, but increased IL-10 expression. In addition, renal neutrophil infiltration was attenuated significantly in the IL-17A^−/−^ septic group. Moreover, IL-17A^−/−^ septic mice showed significantly decreased apoptosis of tubular epithelial cells, including decreased TUNEL-positive tubular cell number and cleaved caspase-3 level, compared with the wild-type CLP group. Their Bax/Bcl-2 expression ratio was also increased.

**Conclusions:**

Our study demonstrates that IL-17A knockout could protect against SA-AKI. We show that IL-17A plays a pathogenic role in SA-AKI by increasing the levels of proinflammatory cytokines and chemokines, and by inducing neutrophil infiltration and apoptosis of tubular epithelial cells. Accordingly, IL-17A may be a novel target in SA-AKI.

**Electronic supplementary material:**

The online version of this article (doi:10.1186/s13613-016-0157-1) contains supplementary material, which is available to authorized users.

## Background

Sepsis-associated acute kidney injury (SA-AKI) is an independent risk factor for death in patients with sepsis [1], but its treatment is limited. To improve the diagnosis and treatment of SA-AKI, we must first understand its pathogenesis. In recent years, the majority of studies on this type of injury have focused on hemodynamic factors after bacterial invasion. However, in many patients, SA-AKI occurs without overt signs of global renal hypoperfusion and SA-AKI has been described in the presence of normal or even increased renal blood flow [2]. Therefore, in recent years, apoptosis, neutrophil infiltration, and the production of proinflammatory cytokines have been considered important mechanisms of SA-AKI [3].

Interleukin (IL)-17 is a proinflammatory cytokine secreted mainly by TH17 cells and γδT cells [4]. It constitutes a novel cytokine family that includes six structurally related isoforms, IL-17A to IL-17F [5]. IL-17A, the first identified member of the IL-17 family, now synonymous with IL-17, acts on a variety of cells through its ubiquitous receptors. In addition, it induces the production of other cytokines and chemokines from a variety of cells and coordinates the recruitment of myeloid cells, such as monocytes and neutrophils, to sites of inflammation [6]. The discovery of the IL-17A cytokine family provided a novel approach to examine the processes underlying inflammatory diseases. It can also help us to identify new therapeutic targets of inflammatory diseases.

Although all clinical research on neutralizing a single mediator of a single target was unsuccessful, the neutralization of IL-17A still showed promise. Studies have reported that IL-17A reached high levels in septic mice after cecal ligation and puncture (CLP) injury, and that the use of antibodies to neutralize IL-17A improved sepsis survival from 10 % to nearly 60 % [7]. Accordingly, targeted IL-17A depletion has been adopted as a therapeutic strategy and is currently undergoing clinical trials for several human autoimmune diseases [8, 9].

Recently, IL-17A has also been shown to be associated with the pathogenesis of kidney and intestine ischemia/reperfusion (I/R) injury [10–12], but its role in SA-AKI remains unclear. Thus, in this study, we used IL-17A^−/−^ mice to evaluate the role of IL-17A in an SA-AKI model induced by CLP injury.

## Methods

### Animals

Male C57BL/6 mice aged 8–10 weeks were purchased from the animal center at The Chinese People’s Liberation Army (PLA) General Hospital (Beijing, China). IL-17A^−/−^ mice in a C57BL/6 background were kindly provided by Professor H. L. Wang (Shanghai Institute of Immunology, Institute of Medical Sciences, Shanghai Jiao Tong University School of Medicine). The mice were maintained on a chow diet in a 12-h light/12-h dark environment at 25 °C at The Chinese PLA General Hospital’s Animal Care Facility, in accordance with institutional guidelines. They were acclimated for 1 week prior to the experiments. All animal studies were approved by the Ethics Committee of The Chinese PLA General Hospital.

### Mouse model of cecal ligation and puncture

Cecal ligation and puncture-induced sepsis was generated as described previously [13]. Mice were anesthetized with 2 % pentobarbital, and a 1- to 2-cm midline incision was made along the linea alba of the abdominal muscle to isolate and exteriorize the cecum. A total of 75 % of the cecum was ligated with a 4-0 silk suture, and the cecum was punctured twice with a 21-gauge needle. A small amount (droplet) of feces was gently extruded from the holes to ensure patency. The cecum was then returned to the peritoneal cavity and the abdominal incision was closed with 4-0 silk sutures. After the operation, 1 mL of pre-warmed normal saline was administered into the peritoneal cavity. In the sham group, mice underwent the same procedure but were neither ligated nor punctured.

Wild-type and IL-17A^−/−^ mice for blood tests and histological and immunochemical analyses were each divided into two groups: a sham group and a CLP group. Then euthanized 24 h after surgery. Wild-type and IL-17A^−/−^ mice for survival study were also divided into two groups: a sham group and a CLP group. Then euthanized 7d after surgery (Additional file 1).

### Survival study

For a survival study, mice in each group were monitored closely for 7 days (*n* = 20/group), after which they were euthanized.

### Assessment of kidney function

Kidney injury was assessed based on the levels of serum creatinine and blood urea nitrogen. Mouse serum samples were obtained at 24 h after CLP injury, and the serum was separated by centrifugation at 3000 rpm for 15 min at 4 °C and then stored at −20 °C until use.

### Histopathology

For histopathological assessment, kidney tissues were harvested 24 h after CLP. Specimens were fixed in 10 % formaldehyde for at least 24 h. The samples were then embedded in paraffin and sectioned at 4-µm thickness for staining with periodic acid-Schiff. Stained sections were observed using a light microscope (Olympus IX71; Olympus, Tokyo, Japan) at a magnification of 400× and evaluated by three independent pathologists. Histological examinations for acute tubular necrosis were semi-quantified and blinded using the following scoring system, as described previously [14]: 0 = none, 1 = ≤10 %; 2 = 11–25 %, 3 = 26–45 %, 4 = 46–75 %, and 5 = >75 %.

### Real-time PCR

Total RNA was extracted from kidney tissue using TRIzol reagent (Invitrogen, Carlsbad, CA, USA) following the manufacturer’s instructions. Reverse transcription was performed using the TaqMan Reverse Transcription Kit (Applied Biosystems, Foster City, CA, USA) and a Gene Amp PCR System 9700 (Applied Biosystems) was used to generate cDNA. Real-time quantitative RT-PCR using 2 μL of cDNA from each sample was performed in duplicate with SYBR Mastermix, in accordance with the manufacturer’s instructions. All reactions were performed using a 7500 Real-time PCR System (Applied Biosystems). For analysis, glyceraldehyde 3-phosphate dehydrogenase (GAPDH) was used as a normalization control. The relative target mRNA expression normalized to GAPDH expression was analyzed using the 2^−ΔΔCT^ method. The primers used are shown in Table 1.

**Table 1:** Primers used for real-time PCR

Gene	Forward primers (5′–3′)	Reverse primer (5′–3′)
IL-17	TGGACTCTCCACCGCAATG	TGGGGGTTTCTTAGGGGTCA
IL-6	AGTCAATTCCAGAAACCGCTATG	ACAGGTCTGTTGGGAGTGGT
TNF-α	CTACTGAACTTCGGGGTGAT	CAGGCTTGTCACTCGAATT
IL-10	AGGCAGCCTTGCAGAAAAGA	GCTCCACTGCCTTGCTCTTA
IFN-γ	ATGAACGCTACACACTGCATC	CCATCCTTTTGCCAGTTCCTC
CXCL1	TGGCTGGGATTCACCTCAAGAACA	TGTGGCTATGACTTCGGTTTGGGT
CXCL2	GAAGTCATAGCCACTCTCAAGG	TTCCGTTGAGGGACAGCA
CXCL5	CGTAACTCCAAAAATTAATCCCAAA	CGAGTGCATTCCGCTTAGCT

### Immunofluorescence studies of kidney samples

Neutrophil granulocyte infiltration into the kidney was evaluated by the detection of Ly6G-positive cells by immunofluorescence; Five-µm-thick kidney sections were cut and incubated overnight with rat anti-Ly6G (1:100; Santa Cruz Biotechnology, Santa Cruz, CA, USA) antibody in a humidified chamber. Secondary antibodies conjugated to CY3 rabbit anti-rat immunoglobulin G (IgG; 1:50; Jackson ImmunoResearch Laboratories, West Grove, PA, USA) were applied for 1 h at room temperature in a darkened humidified chamber. Nuclei were stained with 4′,6-diamidino-2-phenylindole (Zhongshan Goldenbridge Biotechnology, Beijing, China). Each tissue section was observed in a blinded manner under a confocal laser scanning microscope (Olympus FluoView 1000) at a magnification of 600×.

### TUNEL staining

For terminal deoxynucleotidyl-transferase-mediated dUTP nick-end labeling (TUNEL) staining, kidneys were fixed in 4 % paraformaldehyde, embedded in paraffin, and cut into 5-µm-thick sections and stained using the DeadEnd Fluorometric TUNEL system (Promega Corporation, Madison, WI, USA), in accordance with the manufacturer’s instructions, followed by visualization under an Olympus laser scanning confocal microscope (IX81). Quantitative analysis of TUNEL-positive cells was performed using the Image-Pro software in 20 random fields for each mouse by a technician who was blinded to the treatment groups. The mean of all counts was used for comparisons among the groups.

### Western blot analysis

Kidney lysates were prepared by homogenization on ice for 30 min using radioimmunoprecipitation assay buffer [50 mM Tris–Cl (pH 7.6), 150 mM NaCl, 1 % NP-40, 0.1 % sodium dodecyl sulfate, 0.5 % deoxycholic acid, 1 μg/mL leupeptin, 1 μg/mL aprotinin, and 0.5 mM phenylmethylsulfonyl fluoride], and then centrifuged at 12,000 rpm for 30 min at 4 °C, after which supernatants were collected. A total of 100 μg of protein was separated by 15 % sodium dodecyl sulfate–polyacrylamide gel electrophoresis and transferred to a nitrocellulose membrane. Anti-Bax, -Bcl-2, and -cleaved caspase-3 antibodies (Cell Signaling Technology, Danvers, MA, USA) were used as primary antibodies at 4 °C overnight, and horseradish peroxidase-conjugated anti-rabbit/mouse IgG (Santa Cruz Biotechnology) was used as the secondary antibody. Densitometry was performed using Quantity One software (Bio-Rad Laboratories, Hercules, CA, USA).

### Statistics

The survival of mice was estimated by Kaplan–Meier analysis. A log-rank test was used to analyze the survival data. Data are expressed as mean ± standard error. Differences were evaluated using unpaired Student’s *t* tests between two groups and one-way ANOVA for multiple comparisons, followed by a post hoc Student–Newman–Keuls test when necessary. The value of *α* was corrected by the number of comparisons [2*α*/*n* (*n* − 1)] to ensure *α* = 0.05 [15]. mRNA levels were calculated using the 2^−ΔΔCT^ method. Analyses were performed using SPSS for Windows (version 10.1; SPSS, Chicago, IL, USA). Statistical significance was set at *P* < 0.05.

## Results

### Effect of IL-17A knockout on the survival of CLP-induced septic mice

**Fig. 1:**
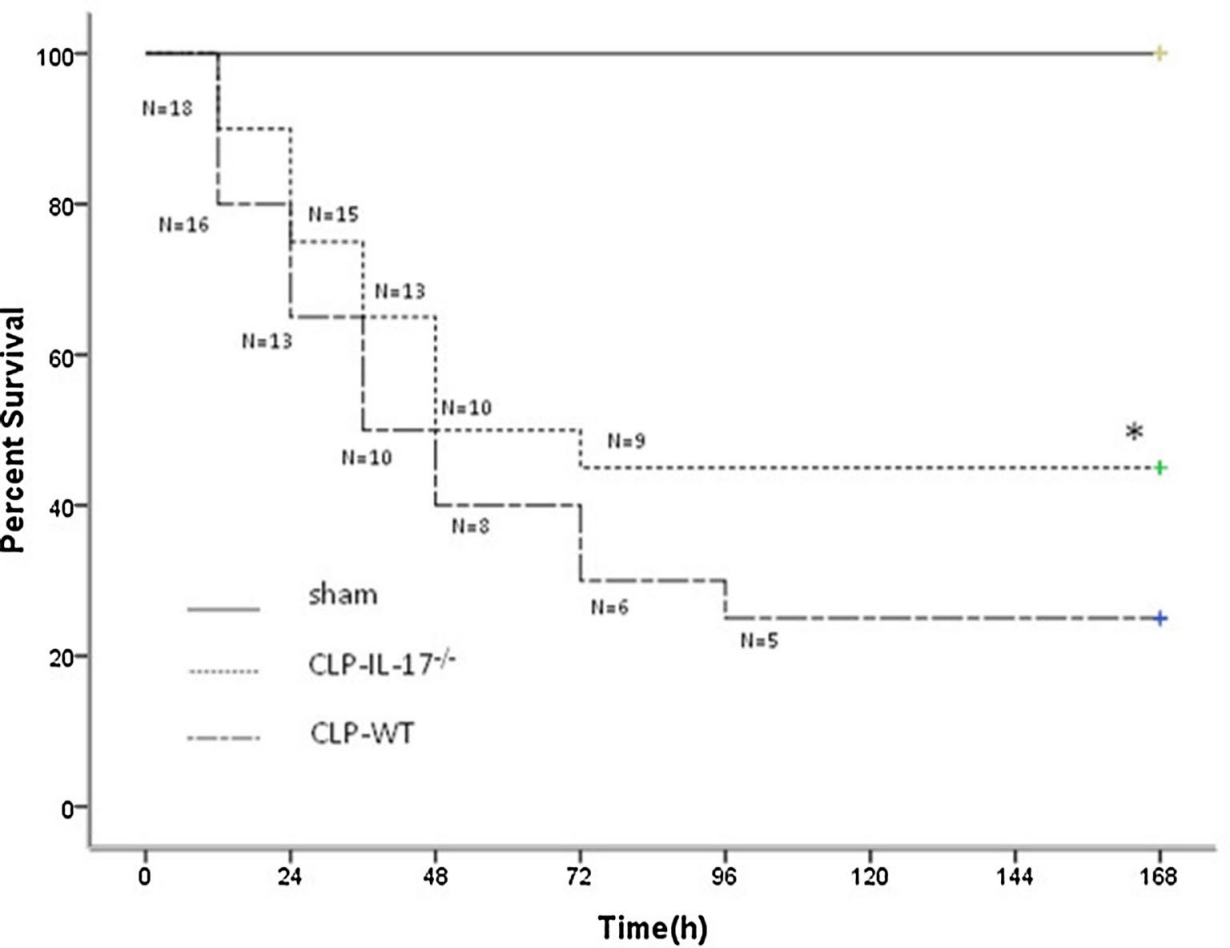
Survival rate of cecal ligation and puncture (CLP) model mice. Each group consisted of 20 animals. Kaplan–Meier *curves* show the survival rate in each group. The survival rate at 7 days was significantly higher in the interleukin (IL)-17A knockout CLP group than in the wild-type CLP group; *P < 0.05

In the absence of antibiotic therapy, we investigated whether IL-17A^−/−^ septic mice showed any improvement in terms of survival at 7 days. At 24 h, seven mice had died in the wild-type CLP group, while only five mice had died in the IL-17A^−/−^-CLP group. At 7 days, the survival rate of the wild-type septic group was 25 %, while that of the knockout IL-17A group was 45 % (Fig. 1). IL-17A knockout significantly improved the survival rate at 7 days compared with that in wild-type mice (*P* < 0.05). In addition, no mortality was observed within 7 days in all sham mice (survival rate = 100 %).

### Effects of IL-17A knockout on renal function in SA-AKI

**Fig. 2:**
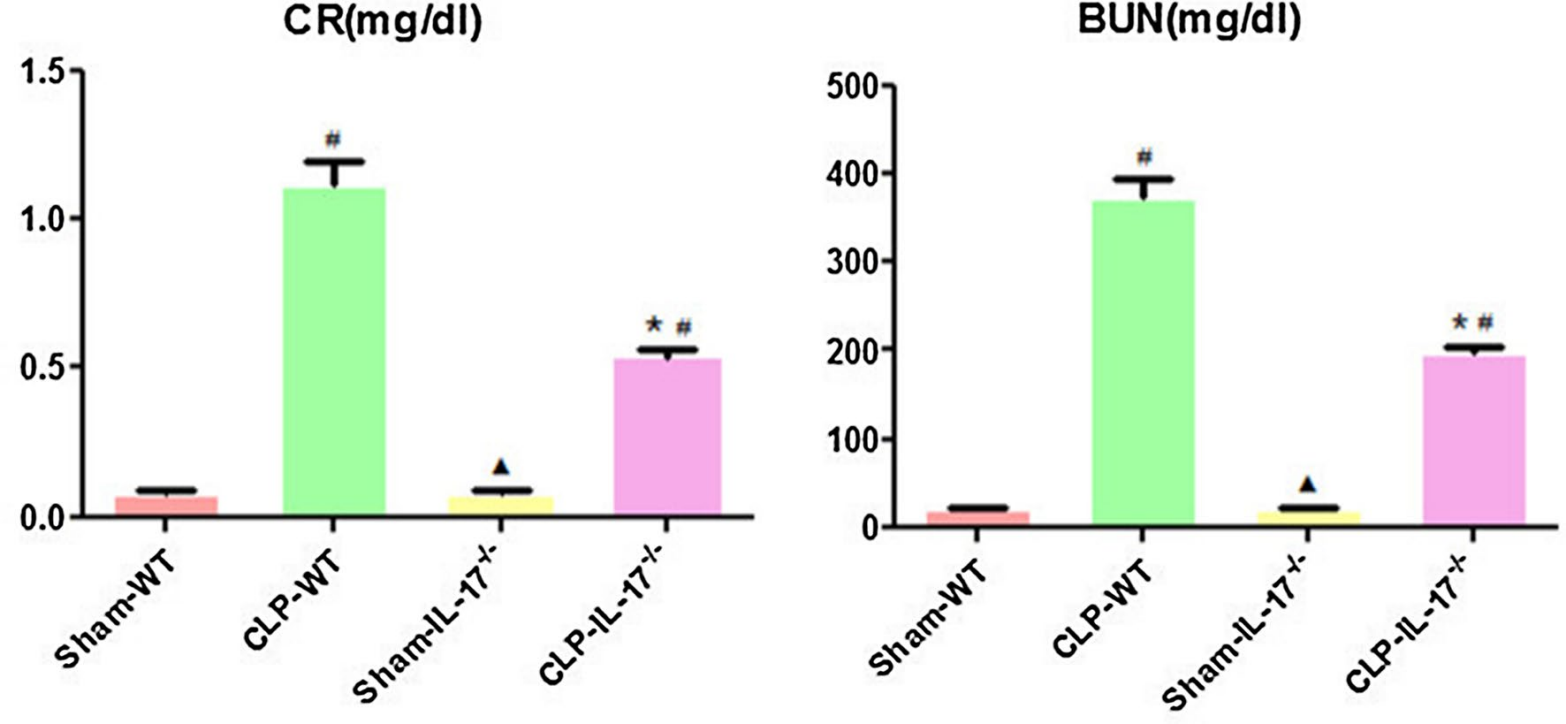
Changes in creatinine and blood urea nitrogen in the four groups. IL-17 knockout significantly reduced creatinine (Cr) and blood urea nitrogen (BUN) levels 24 h after CLP injury compared with the levels in wild-type CLP mice. ^#^ *P* < 0.05 compared with the sham group. *P < 0.05 compared with the wild-type CLP group. Wild-type sham group *n* = 20, IL-17A^−/−^ sham group *n* = 20, wild-type CLP group *n* = 1 2, and IL-17A^−/−^ septic group *n* = 15

Renal injury was assessed by measuring blood urea nitrogen and creatinine levels at 24 h. Baseline levels of these plasma markers of kidney injury did not differ significantly between the wild-type and IL-17A^−/−^ sham groups. Levels of blood urea nitrogen and creatinine were increased in wild-type and IL-17A^−/−^ mice with CLP-induced sepsis compared with those in the sham group, indicative of the development of SA-AKI. Compared with wild-type septic mice, IL-17A^−/−^ septic mice showed significantly reduced creatinine and blood urea nitrogen levels (*P* < 0.05; Fig. 2).

### Effects of IL-17A knockout on renal histology in SA-AKI

**Fig. 3:**
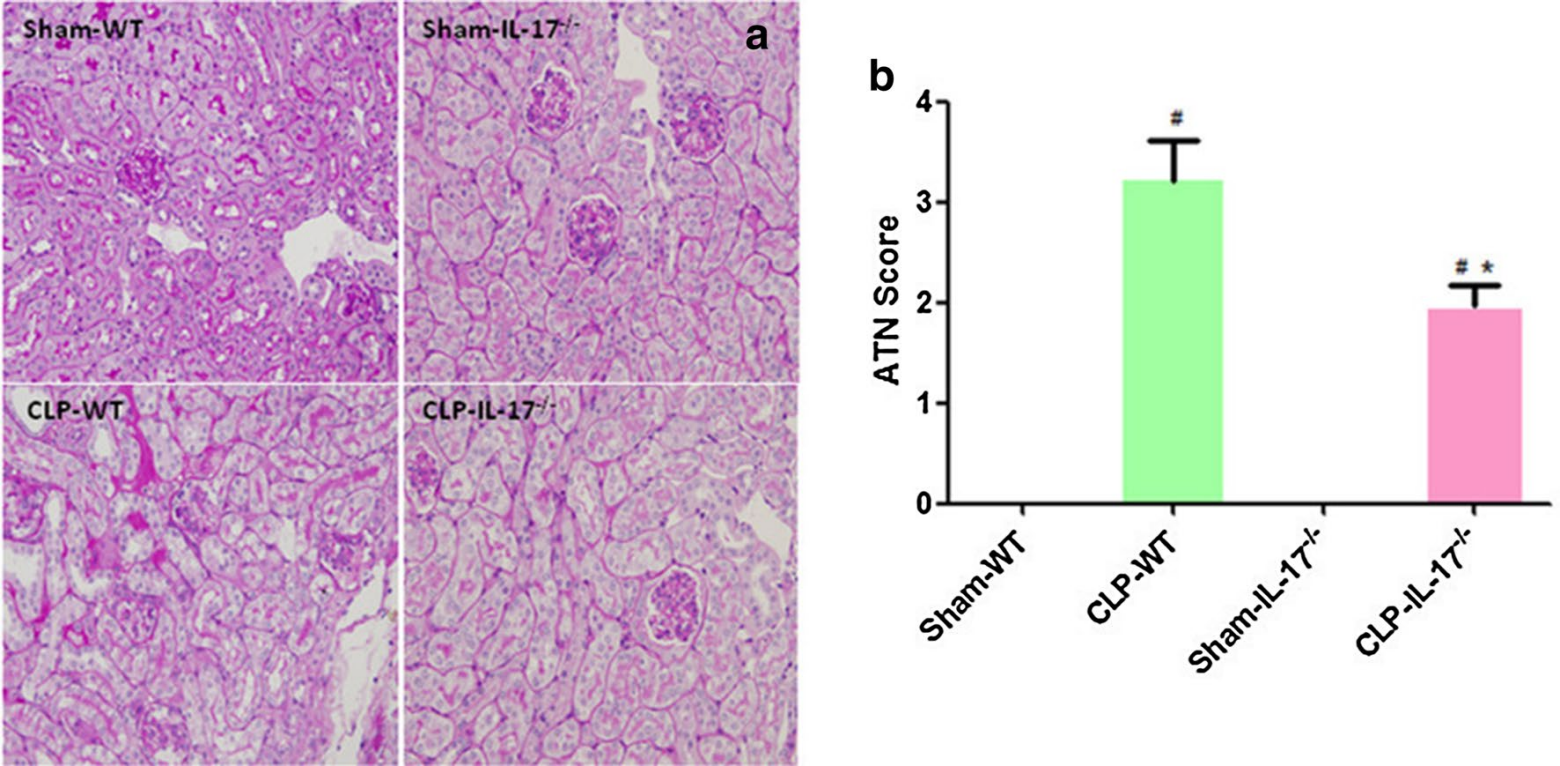
Renal histology changes in the four groups. **a** Sections were subjected to Periodic acid/Schiff staining to assess kidney morphology. **b** Semi-quantitation of the morphological changes using a histological grading system. The data are presented as mean ± standard deviation (SD). ^#^ *P* < 0.05 compared with the sham group. *P < 0.05 compared with the wild-type CLP group. Wild-type sham group *n* = 20, IL-17A^−/−^ sham group *n* = 20, wild-type CLP group *n* = 12, and IL-17A^−/−^ septic group *n* = 15

Morphological changes in the sepsis group were scored 24 h after CLP injury based on brush border loss, tubular degeneration, and vacuolization in the proximal tubules. Knockout of IL-17A ameliorated the tissue damage and reduced the tubular injury score (*P* < 0.05; Fig. 3). Taken together, our data show that CLP-induced polymicrobial sepsis resulted in SA-AKI, the severity of which was reduced by IL-17A knockout.

### Effect of IL-17A knockout on renal cytokine and chemokine mRNA levels

**Fig. 4:**
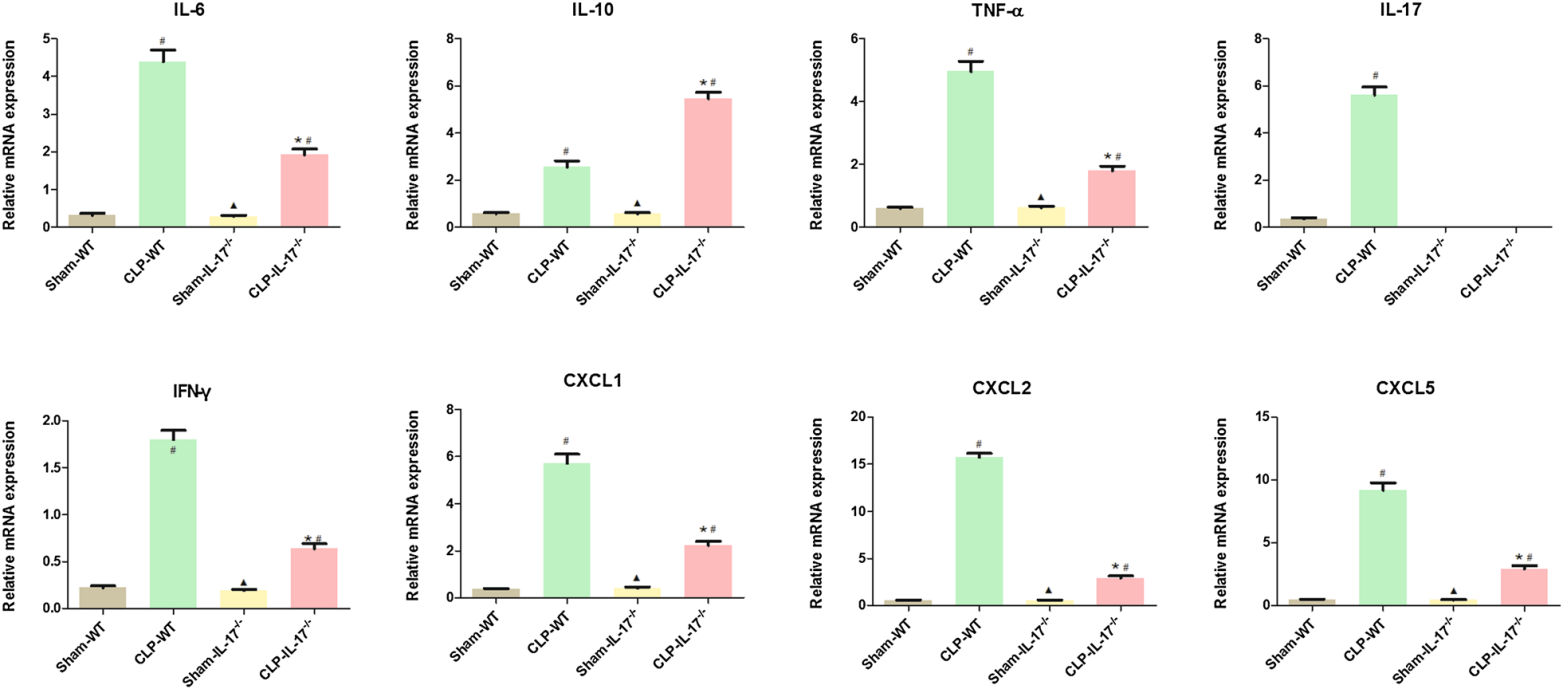
mRNA levels of inflammatory cytokines and chemokines in the kidneys. The mRNA levels of the inflammatory cytokines IL-10, IL-17, IL-6, interferon-γ, and tumor necrosis factor-α and the chemokines CXCL1, CXCL2, and CXCL5 in kidney tissue 24 h after sham or CLP operation. ^#^ *P* < 0.05 compared with the sham group. *P < 0.05 compared with the wild-type CLP group. Wild-type sham group *n* = 20, IL-17A^−/−^ sham group *n* = 20, wild-type CLP group *n* = 12, and IL-17A^−/−^ septic group *n* = 15

Previous studies showed that IL-17A in peritoneal fluid plays a critical role during severe polymicrobial sepsis [7]. We hypothesized that IL-17A would play an important role in SA-AKI. To investigate whether IL-17A knockout affected local renal production of inflammatory cytokines and chemokines, we assayed the renal mRNA levels of these cytokines using RT-PCR. The IL-17A, IL-6, interferon (IFN)-γ, tumor necrosis factor (TNF)-α, and IL-10 mRNA levels were increased significantly in both CLP groups at 24 h (Fig. 4). Compared with the wild-type septic group, IL-17A knockout decreased the IL-17A, IL-6, IFN-γ, and TNF-α mRNA levels (*P* < 0.05), but increased the level of IL-10 (*P* < 0.05; Fig. 4).

The chemokines CXCL1, CXCL2, and CXCL5 are not only potent neutrophil chemoattractants but are also encoded by IL-17A target genes. We thus examined the mRNA levels of these chemokine genes related to IL-17A function in the kidney using RT-PCR. The CXCL1, CXCL2, and CXCL5 mRNA levels were increased significantly in the wild-type CLP group and decreased in the IL-17A^−/−^ septic group (*P* < 0.05; Fig. 4). SA-AKI caused a significant increase in the mRNA levels of all five chemokines, consistent with increased neutrophil infiltration. Moreover, IL-17A knockout had the opposite effect on the expression of these chemokines after SA-AKI.

### Effect of IL-17A knockout on neutrophil infiltration

**Fig. 5:**
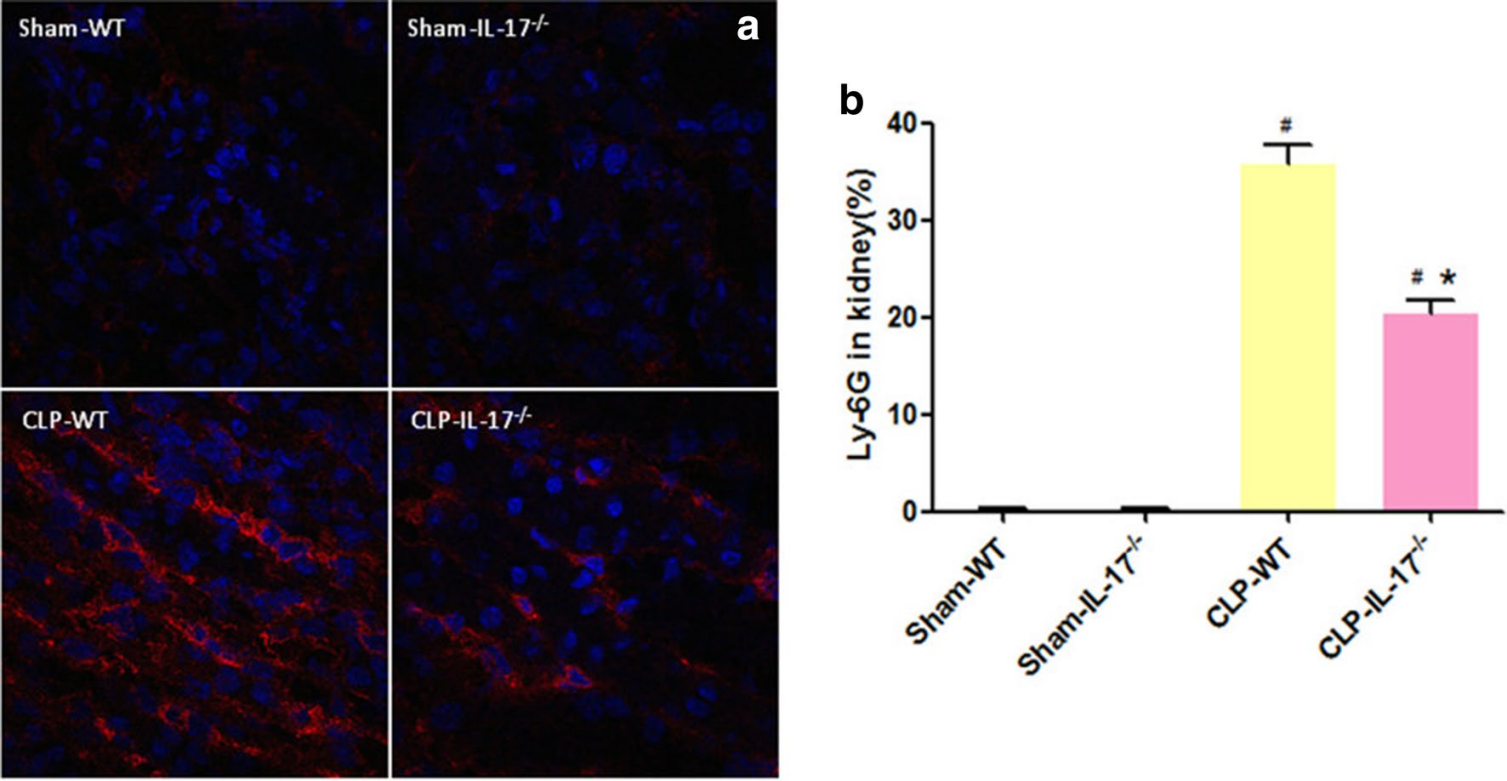
Infiltration of neutrophils in the kidneys of the four groups. Histological sections of kidneys were subjected to immunofluorescence staining, and the number of positive cells was counted in five high-powered fields per section (original magnification ×400). Values represent mean ± SD. ^#^ *P* < 0.05 compared with the sham group. *P < 0.05 compared with the wild-type CLP group. Wild-type sham group *n* = 20, IL-17A^−/−^ sham group *n* = 20, wild-type CLP group *n* = 12, and IL-17A^−/−^ septic group *n* = 15

Neutrophil infiltration is a hallmark of inflammatory injury after sepsis [16], and one of the main functions of IL-17A is neutrophil recruitment. To determine whether neutrophil infiltration played a role in our model, we examined the degree of neutrophil infiltration in the kidney using immunofluorescence. No Ly-6G-positive cell infiltration was detected in the sham group of wild-type and IL-17A knockout mice (Fig. 5). In the wild-type septic group, many Ly-6G-positive cells were observed in the renal tissue (Fig. 5), primarily in the renal cortex, including the glomerulus and tubulointerstitium. Compared with the wild-type septic group, the IL-17A^−/−^ septic group exhibited less neutrophil infiltration (Fig. 5).

### Anti-apoptotic effects of IL-17A knockout in SA-AKI

**Fig. 6:**
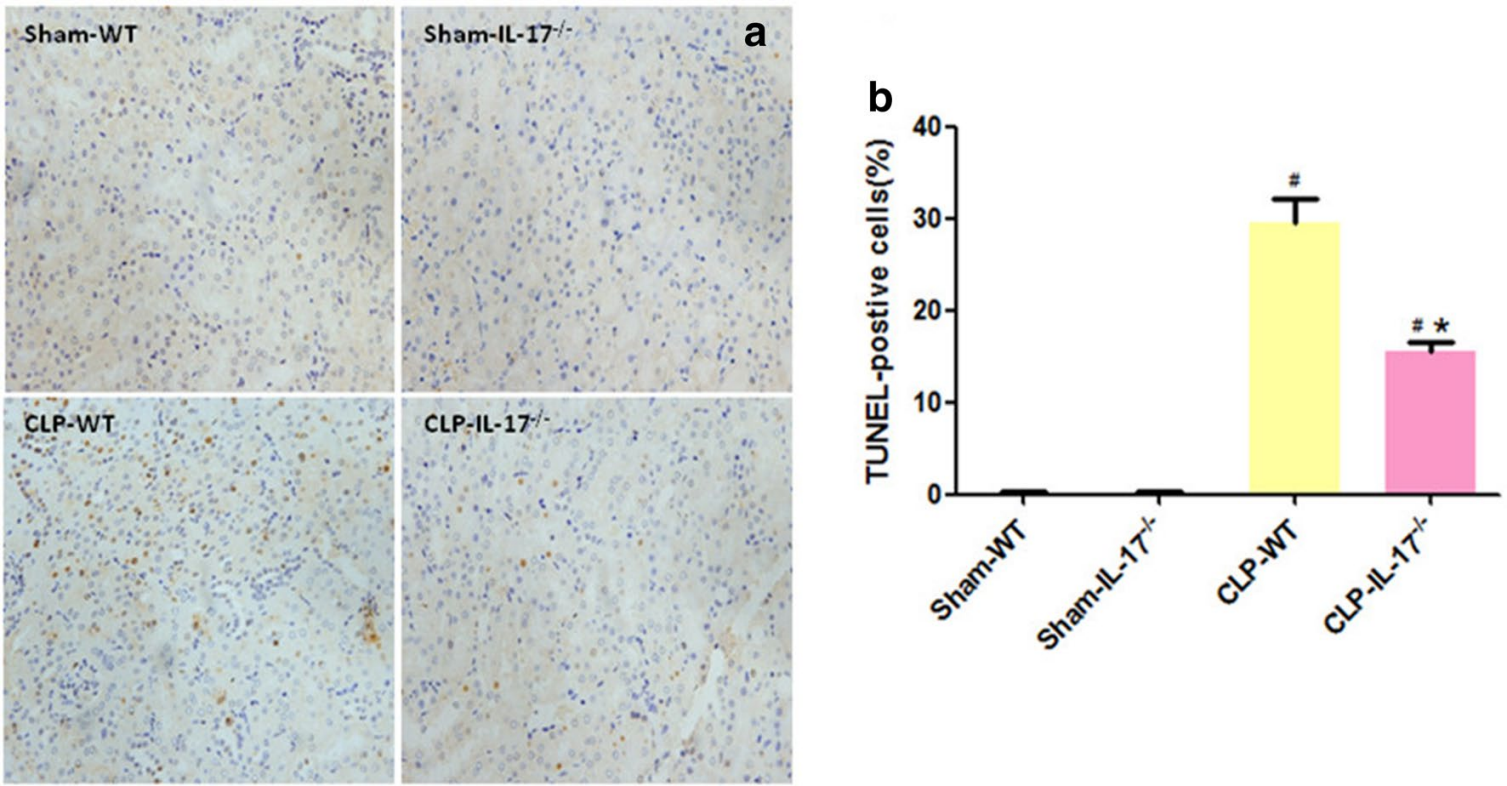
Numbers of terminal deoxynucleotidyl-transferase-mediated dUTP nick-end labeling (TUNEL)-positive cells in the kidneys. Quantitative analysis of TUNEL-positive cells from 20 random fields per mouse. The data are presented as mean ± SD. ^#^ *P* < 0.05 compared with the sham group. *P < 0.05 compared with the wild-type CLP group. Wild-type sham group *n* = 20, IL-17A^−/−^ sham group *n* = 20, wild-type CLP group *n* = 12, and IL-17A^−/−^ septic group *n* = 15

Apoptosis contributes significantly to AKI. We hypothesized that the role of IL-17A in SA-AKI is associated with tubular apoptosis. To test this hypothesis, we performed TUNEL assays of renal sections obtained 24 h after CLP injury. As shown in Fig. 6, IL-17A knockout significantly decreased the number of TUNEL-positive tubular cells compared with that in wild-type CLP mice, suggesting that IL17A knockout reduced the extent of tubular apoptosis.

**Fig. 7:**
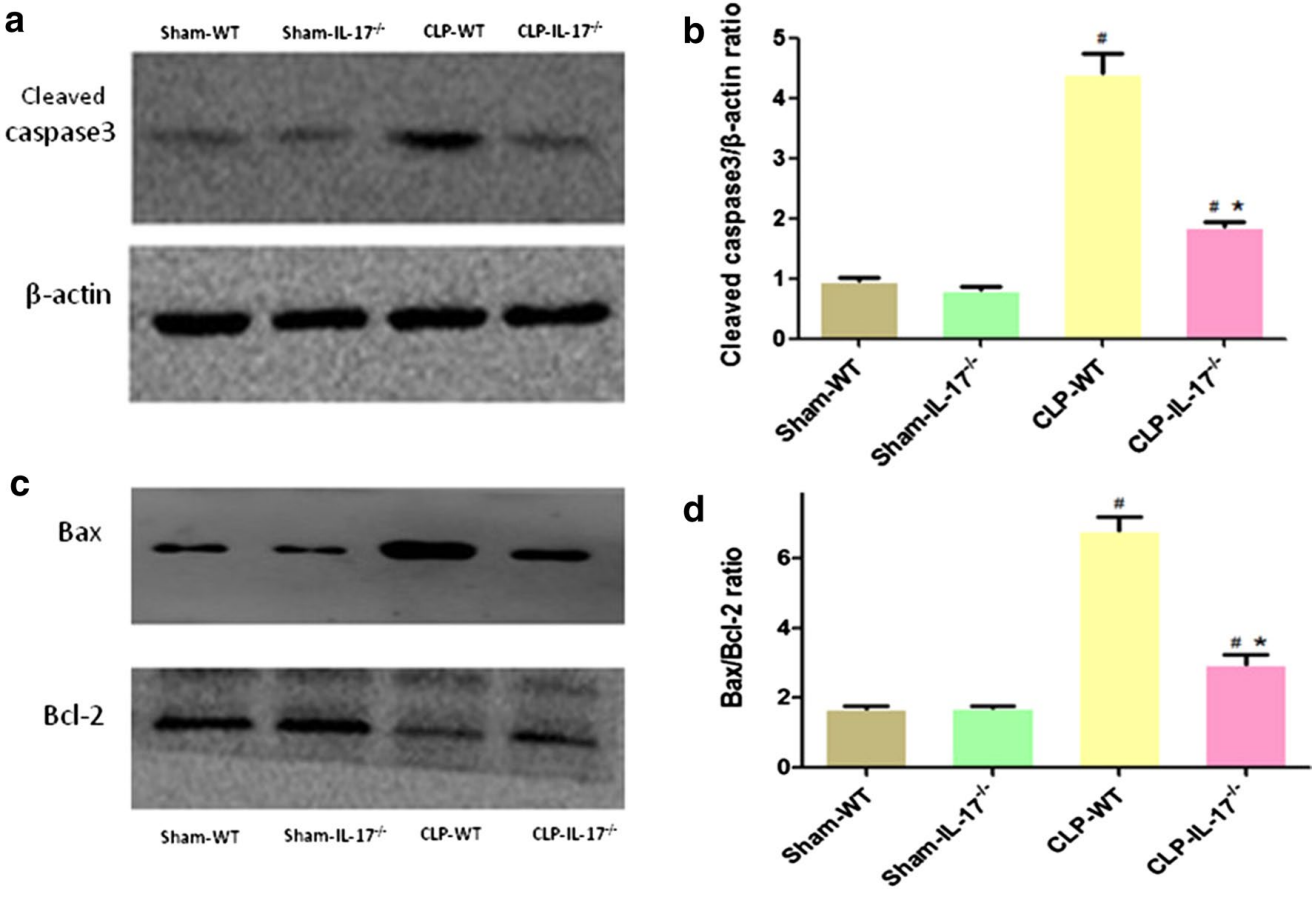
Expression of Bax, Bcl-2, and cleaved caspase-3 in the kidneys. **a** Western blot results for cleaved caspase-3 protein. **b** Quantitative analysis of cleaved caspase-3. **c** Western blot of Bax and Bcl-2 proteins. **d** Quantitative analysis of Bax and Bcl-2. The protein levels are presented as mean ± SD. ^#^ *P* < 0.05 compared with the sham group. *P < 0.05 compared with the wild-type CLP group. Wild-type sham group *n* = 20, IL-17A^−/−^ sham group *n* = 20, wild-type CLP group *n* = 12, and IL-17A^−/−^ septic group *n* = 15

The expression of cleaved caspase-3, a biomarker of apoptosis, was increased significantly in the wild-type septic group (Fig. 7). The IL-17A^−/−^ septic group also showed an increased cleaved caspase-3 level, albeit significantly less than that in the wild-type septic group (Fig. 7). Furthermore, Bax expression was increased significantly in the wild-type septic group, while there was less expression in the kidney of IL-17A^−/−^ septic mice at the same time (Fig. 7). In addition, Bcl-2 expression decreased slightly in both septic groups. The Bax/Bcl-2 ratio was increased to a greater extent in the wild-type septic group than in the IL-17A^−/−^ septic group. Therefore, IL-17A knockout attenuated the occurrence of kidney cell death induced by CLP injury.

## Discussion

In recent years, research has revealed that the main pathogenic factors in SA-AKI are not hemodynamic failure or ischemia, as previously thought, but inflammation and apoptosis [3]. Accordingly, therapies aimed at suppressing apoptosis and inflammation are currently being sought.

In our study, we revealed that IL-17A knockout caused a marked amelioration of SA-AKI, which was associated with a reduction in neutrophil infiltration and tubular cell apoptosis. Moreover, IL-17A enhanced neutrophil infiltration, possibly by inducing CXC chemokine-mediated neutrophil migration. In light of these novel findings, IL-17A may become a new therapeutic target.

Increasing evidence has indicated that both adaptive and innate immunity play a role in the pathogenesis of sepsis [17]. Notably, IL-17A acts as a bridge between adaptive and innate immunity through the inflammatory response [18], and thus occupies a unique position in the pathogenic process of sepsis. A growing body of evidence has also demonstrated that IL-17A is involved in the inflammatory response during kidney injury in acute renal obstruction [19]. However, little was previously known about SA-AKI, although IL-17A is involved in the pathogenesis of sepsis. In this context, we previously reported the important finding that circulating IL-17A levels were elevated in SA-AKI [20]. Therefore, we focused on the effect of IL-17A in SA-AKI using the IL-17A^−/−^ CLP mouse model. Notably, in IL-17A^−/−^ mice, kidney function and morphology were found to be protected after SA-AKI. In this study, genetic deficiency in IL-17A ameliorated SA-AKI markedly, as demonstrated by reduced mortality, improved inflammatory markers, and reduced neutrophil infiltration, which demonstrated a critical role for IL-17A in SA-AKI.

Neutrophil recruitment plays a major role in SA-AKI. Neutrophil chemotaxis and activation may be regulated by CXC chemokines. Interestingly, IL-17A has also been shown to induce the production of CXCL1, CXCL2, and CXCL5 in mouse osteoblastic cells and lung fibroblasts [21, 22]. Moreover, IL-17A has been shown to induce the recruitment of neutrophils into the airways via the production of CXC chemokines [23]. However, the production of IL-17A during infection has not always been correlated with protection. In a murine CLP model of polymicrobial sepsis, IL-17A from γδT cells was detected, but its depletion led to a decrease in bacteremia and reductions in systemic proinflammatory cytokines (TNF-α, IL-1β, and IL-6) and chemokines [24]. In our study, we found that the inhibition of IL-17A markedly decreased renal CXCL1, CXCL2, and CXCL5 expression and neutrophil infiltration. This is consistent with previous reports describing that elevated IL-17A induced neutrophil recruitment and increased inflammation in a more severe CLP model [7]. IL-17A also enhanced cardiomyocyte LIX, KC, and MIP-2 production and neutrophil migration to the supernatant from conditioned cardiomyocytes in vitro [25]. Although we have no further data to validate the pathogenic role of IL-17A in SA-AKI in vitro, which is a limitation of our study, our results still indicate that IL-17A may contribute to SA-AKI, at least in part through the regulation of neutrophil infiltration to the kidney by affecting CXC chemokine expression.

Apoptosis plays an important role in health and disease. It also contributes to kidney dysfunction in I/R or nephrotoxin-induced AKI [26, 27]. However, no consensus has been reached on the role of renal cell apoptosis in the development of SA-AKI. In terms of the histopathological findings from biopsies taken from septic AKI patients, these have been reported to include acute tubular injury or necrosis to varying degrees; leukocyte infiltration into glomeruli, interstitial capillaries, and tubular lumen; and apoptosis in tubular cells and on rare occasions in glomerular cells [28, 29]. In contrast, Langenberg et al. [30] reported a lack of any tubular injury or apoptosis in a sheep SA-AKI model. However, in our study, we observed that a substantial number of tubular cells underwent apoptosis. In addition to increased numbers of TUNEL-positive cells, cleaved caspase-3 activity and Bax were also increased in septic kidneys compared with those in sham kidneys. Meanwhile, the reduced level of the anti-apoptosis protein Bcl-2 also suggested that apoptosis occurred in the kidneys of septic mice after CLP surgery. IL-17A has also been shown to induce apoptosis of vascular smooth muscle cells and airway epithelial cells [31, 32]. However, there are no reports on the role of IL-17A in renal tubular cell apoptosis. Our in vivo study showed that IL-17A knockout reduced tubular cell apoptosis, as confirmed by the decreased number of TUNEL-positive tubular cells, the reduced cleaved caspase-3 activity, and the ratio of pro-apoptotic (Bax) to anti-apoptotic (Bcl-2) proteins. IL-17A may trigger apoptosis directly in tubular cells or indirectly by mediating the production of other factors. However, the regulation of cleaved caspase-3 activity and the Bax/Bcl-2 ratio demonstrated that IL-17A activated intrinsic signaling pathways. These findings indicate that IL-17A plays a pathogenic role in SA-AKI by inducing apoptosis and neutrophil infiltration (Additional file 1).

There are some limitations to this study. A previous study reported that SA-AKI does not result from hypoperfusion alone, but may arise to a large extent from renal inflammation and tubular responses to various sepsis mediators. In many patients, SA-AKI occurs without overt signs of global renal hypoperfusion and SA-AKI has been described in the presence of normal or even increased renal blood flow [33]. Therefore, in our study, we did not perform hemodynamic monitoring, which was one of the limitations. Another previous study also reported that the neutralization of peritoneal IL-17A markedly improves lung injury in severe CLP-induced septic mice [7]. Although we did not prove a beneficial effect of blocking IL-17A in other organ systems in this study, based on the above data, we believe that the beneficial effect of blocking IL-17A could be mediated by other organ systems as well as AKI. Moreover, although we observed decreased levels of IL-17A-related cytokines and chemokines in renal tissues after IL-17A knockout, the mechanism by which IL-17A knockout could protect against SA-AKI remains unclear. It is also needs to be determined whether the improved renal apoptosis is directly related to the knockout of IL-17A.

## Conclusions

Overall, our study demonstrated that IL-17A knockout could protect against SA-AKI. We showed that IL-17A may play a pathogenic role in SA-AKI by increasing the levels of proinflammatory cytokines and chemokines, and reducing neutrophil infiltration and apoptosis of tubular epithelial cells. Accordingly, IL-17A may be a novel target in the treatment of SA-AKI.

## Additional file

10.1186/s13613-016-0157-1 The grouping of experimental animals.

## Abbreviations

AKI: acute kidney injury
IL-17(A): interleukin 17(A)
CLP: cecal ligation and puncture
SA-AKI: sepsis-associated acute kidney injury
ANOVA: one-way analysis of variance
