# Supplementary figures and images for: Knockout of interleukin-17A protects against sepsis-associated acute kidney injury
Source: Ann Intensive Care. 2016 Jun 22;6:56. doi: 10.1186/s13613-016-0157-1 (PMC4917508; doi:10.1186/s13613-016-0157-1)

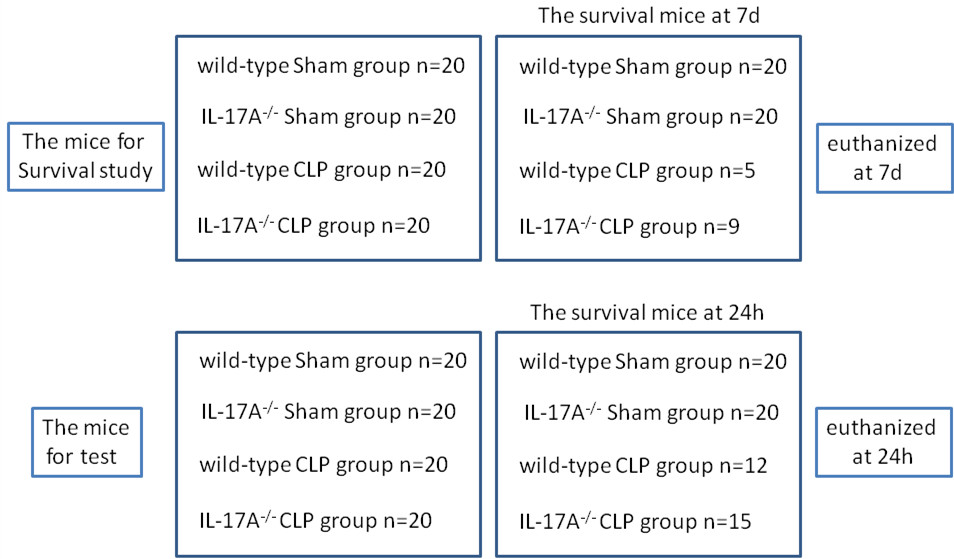

Supplement: Supplementary file 1 — 10.1186/s13613-016-0157-1 The grouping of experimental animals. [file 13613_2016_157_MOESM1_ESM.jpg]
